# Fe-N-Doped Conjugated Organic Polymer Efficiently Enhanced the Removal Rate of Cr(VI) from Water

**DOI:** 10.3390/polym15132918

**Published:** 2023-06-30

**Authors:** Cheng Tang, Tao Hu, Chengzhen Du, Ziqin Liao, Wenyan Cheng, Fen Wang, Xiaoli Hu, Kunpeng Song

**Affiliations:** 1 Key Laboratory of Low-Cost Rural Environmental Treatment Technology, Education Department of Sichuan Province, Sichuan University of Arts and Science, No. 406, Nanbin Road, 3rd Section, Dazhou 635000, China; 2 Chemical Synthesis and Pollution Control Key Laboratory of Sichuan Province, College of Chemistry and Chemical Engineering, China West Normal University, Shida Road, Nanchong 637009, China

**Keywords:** organic polymers, Fe-N co-doping, Cr(VI) removal, conjugate structure

## Abstract

A Fe-N conjugated organic polymer (SMP-Fr-Py) was prepared from ferrocene and pyrrole using a Scholl coupling reaction, which significantly improved the performance of Cr(VI) removal compared to the polymer (HCP-Fr-Py) prepared by adding the cross-linker formaldehyde dimethyl acetal (FDA). The results showed that at a pH of 2 and at 25 °C, the removal of Cr(VI) reached 90% for SMP-Fr-Py and only 58% for HCP-Fr-Py after 20 min of reaction. Subsequently, 99% and 78% were achieved after 120 min of reaction, respectively. The test results showed that the removal reaction followed a pseudo-second-order kinetic model. The removal efficiency decreased with increasing solution pH and initial Cr(VI) concentration, but increased with increasing SMP-Fr-Py dosage, reaching three cycles. The characterization of the reaction complexes and measurements of Cr species conversion revealed the near absence of Cr(VI) species in the solution. Approximately 38% of Cr(VI) was found to be adsorbed on the material surface, with another fraction present in solution (24%) and on the material surface (38%) in the form of Cr(III). The overall study showed that the direct connection of ferrocene and pyrrole in SMP-Fr-Py through C-C bonding increased the conjugated structure of the polymer backbone, which facilitated electron transfer and transport. Furthermore, the Fe-N elements worked synergistically with each other more easily, which improved the removal performance of Cr(VI) and provided a reference for the subsequent work.

## 1. Introduction

Cr has a wide range of applications in industrial production, including metal smelting, textiles, pigments, electroplating, and as catalysts [1,2]. The risks of Cr to human health and ecosystems are related to the chemical form in which it is encountered [3,4,5]. Cr(III) is usually present in the environment as Cr(OH)_3_, and (CrFe) (OH)_3_ precipitates with low solubility and poor mobility in aqueous environments [6]. Conversely, Cr(VI) is more soluble than Cr(III) and more toxic to microorganisms, plants, animals, and humans, and is listed as one of the top 20 hazardous substances in the Agency for Toxic Substances and Disease Registry (ATSDR). The sources of Cr pollution include the processing of Cr-containing ores, metal surface treatment and dyeing, etc. Therefore, the removal of Cr(VI) from wastewater is necessary [7,8,9]. Several reported methods for Cr(VI) removal are mainly physical (adsorption, membrane separation, and ion exchange methods), chemical (chemical precipitation, flocculation/coagulation, and redox methods), and biological (bacterial organisms, fungal organisms, algal organisms, and plant restoration) [10,11,12]. Among them, the complex and costly process of the physical method and the time-consuming biological method greatly hinder the application of these techniques in Cr(VI) treatment. On the contrary, chemical methods are receiving more and more attention because of their simple processes and high efficiency, which have great potential for Cr(VI) removal [13,14].

One common remediation technique for Cr(VI) contamination is chemical reduction, which involves reducing Cr(VI) to the less toxic and biocompatible Cr(III) [15,16,17]. Emerging research has focused on the development of nanomaterials that possess both adsorption and reduction capabilities as a means of effectively removing Cr(VI) from contaminated environments [18,19]. Iron materials, such as nanoscale zero-valent iron (nZVI) [20], iron oxides [21,22], and iron sulfides [23,24], are commonly used for the reduction or adsorption of Cr(VI) [25,26,27,28]. Furthermore, a large number of Fe-based composites have been developed for the removal of Cr(VI) from water. For example, Dong and colleagues demonstrated that biochar was a good conductor and that electrons from Fe^0^ nuclei were transferred to Cr(VI) via acid treated biochar (HCl-BC). It was confirmed that the main active component of nZVI@HCl-BC particles was nZVI, which acts as a reducing agent, whereas HCl-BC only acts as an electron transfer medium and carrier [3]. The results illustrate that nano-zero-valent iron and its composites readily produce reducing species, such as H•, hydrogen gas, and ferrous ions, in acid solutions, and these intermediates are direct electron donors for the reduction of Cr(VI) [14,29]. Although nZVI and its composites have a good ability to remove Cr(VI), they also tend to produce sludge containing large amounts of iron and chromium, causing secondary pollution [30,31]. Therefore, it is necessary to consider more precise uses of metallic iron and its compounds, as well as better immobilization methods for metals and their corresponding compounds.

Our previous work has demonstrated that organic polymers containing iron functional groups have some ability to remove Cr(VI). These materials can be synthesized in a one-step process through the post-cross-linking of aromatic compounds containing metal functional groups [32]. Ferrocene, an organic transition metal compound with an aromatic nature, is chemically very stable, with the central iron atom maintaining an oxidation state of +2 valence. There are not many applications of ferrocene itself, but a wide variety of derivatives have been synthesized to expand the range of applications for ferrocene [33,34,35]. At present, there are few studies focusing on the direct use of ferrocene synthetic composites for the removal of Cr(VI) [36,37].

**Scheme 1:**
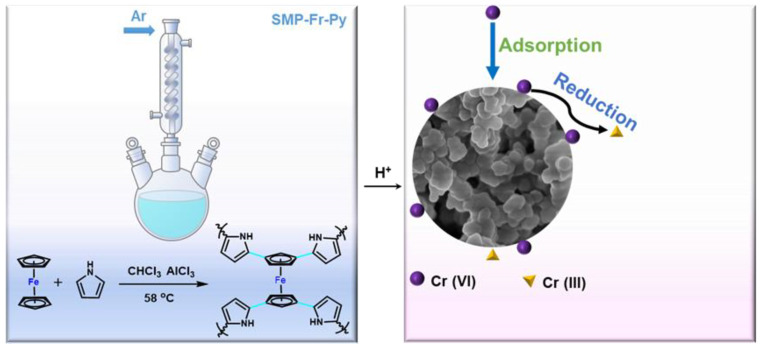
Schematic diagram of the synthetic route of SMP-Fr-Py.

In this work, Fe-N conjugated organic polymers were prepared using a Scholl coupling reaction. SMP-Fr-Py was synthesized under the protection of inert gas using ferrocene and pyrrole as raw materials, chloroform as the solvent, and anhydrous AlCl_3_ as the catalyst (Scheme 1). The polymer HCP-Fr-Py, cross-linked with formaldehyde dimethyl acetal (FDA), was used as comparative material. The physicochemical properties of the polymers were studied using IR, ^13^CNMR, and XPS, and the microscopic morphology and porous properties of the materials were investigated using SEM, TEM, and BET. After this, various process conditions and parameters for the removal of Cr(VI) were investigated, including the initial Cr(VI) concentration, pH value, and contact time, in order to investigate the Cr(VI) removal performance of SMP-Fr-Py in the water system. In order to elucidate the possible mechanism of SMP-Fr-Py for the repair of Cr(VI), adsorption kinetics studies and the analysis of Cr species related to the adsorbent and treated water were performed. Finally, the reusability of the polymer was investigated.

## 2. Materials and Methods

### 2.1. Materials

Ferrocene, pyrrole (steamed), chloroform, formaldehyde dimethyl acetal (FDA), and 1, 2-Dichloroethane (DCE) were purchased from Aladdin Chemical Reagent Co., Ltd. (Fengxian District, Shanghai, China) These were all analytical reagents. Anhydrous ferric chloride, anhydrous aluminum trichloride, and other solvents were purchased from Adamas-beta, and these were all analytical reagents.

### 2.2. Sample Preparation

#### 2.2.1. Synthesis of SMP-Fr-Py

SMP-Fr-Py was synthesized using the Scholl coupling reaction method [38]. Ferrocene (10 mmol, 1.86 g) and pyrrole (10 mmol, 0.67 g) were first added to a 100 mL two-necked flask. This was followed by the addition of CHCl_3_ (20 mL), which was magnetically stirred well, and the addition of anhydrous AlCl_3_ (60 mmol, 8.00 g) to the above solution with continuous stirring for 48 h at 58 °C under Ar protection. The product was washed with methanol until the liquid was light in color. It was then washed twice with HCl-H_2_O (*v*/*v* = 2:1), followed by Soxhlet extraction with methanol for 48 h, and it was then dried in a vacuum oven at 60 °C. Yield: 77%.

#### 2.2.2. Synthesis of HCP-Fr-Py

HCP-Fr-Py was synthesized using the knitting method with FDA as an external cross-linker [39]. Ferrocene (10 mmol, 1.86 g) and pyrrole (10 mmol, 0.67 g) were added to a 100 mL two-necked flask under air atmosphere. This was followed by the addition of DCE (20 mL) with magnetic stirring, and subsequently, FDA (30 mmol, 2.28 g). A condensing reflux device was set up and anhydrous FeCl_3_ (18 mmol, 2.925 g) was added to the above solution. The mixture was heated at 45 °C for 5 h to complete the initial polymerization reaction. Then, it was heated at 80 °C for 19 h with vigorous stirring. After cooling to room temperature at the end of the reaction, the solid product was washed with methanol until the liquid was light in color. Then, the methanol was subjected to Soxhlet extraction for 48 h and was finally dried in a vacuum oven at 60 °C. Yield: 80%.

### 2.3. Characterization

The FT-IR spectra of the samples were recorded using the NEX-US670 spectrometer (Madison, WI, USA) under environmental conditions, with a resolution of 4 cm^−1^ in the wave number range of 4000–400 cm^−1^. Scanning electron microscopy and EDS mapping experiments were conducted using GeminiSEM 500 (Oberkochen, Germany). The transmission electron microscopy (TEM) experiments were performed using a JEM-2100F field emission electron microscope (JEOL, Tokyo, Japan) with an accelerated voltage of 200 kV, including a probe corrector. Solid State Nuclear Magnetic Resonance (SSNMR) ^13^C NMR spectra were recorded using a Brock AVANCE III 600 M device. The specific surface area was calculated using the Brunauer–Emmett–Teller (BET) method, and the aperture distribution was analyzed using the non-local density function theory. The X-ray photoelectron spectroscopy (XPS) analysis was carried out using an ESCALAB 250 spectrometer (Waltham, MA, USA).

### 2.4. Experimental Procedures

A 1000 mg/L Cr(VI) stock solution was prepared. The Cr(VI) stock solution was diluted with deionized water to produce the working solution. Cr(VI) removal was conducted in a 100 mL conical flask under ambient conditions. The typical Cr(VI) removal procedure involved the addition of 0.03 g of material to a 40 mL conical flask containing Cr(VI) solution. The flask was shaken on a rotary shaker at 30 °C. In order to examine the effect of dissolution pH, 0.1 mol/L of HCl or NaOH was used to adjust the initial pH of the Cr(VI) solution. At fixed intervals, samples from the flask were removed using a syringe and were then passed through a 0.22 μm teflon membrane filter. In order to evaluate the stability of SMP-Fr-Py, the reaction solids were separated from the solution, thoroughly washed with deionized water, and dried under vacuum before usage.

### 2.5. Analytical Methods

The concentration of Cr(VI) in aqueous solutions was determined using the 1,5-diphenylcarbazide (DPC) method. A mixture of mixed acid and DPC (color developer) was added to 0.5 mL of filtrate and was incubated for 5 min. The absorbance was measured at 540 nm using a UV-Vis spectrometer (UV-752N INESA, Shanghai, China). The total Cr in the aqueous solution was measured using an ICP-OES 5110 spectrometer (Agilent Technologies, CA, USA). The concentration of Cr(III) was calculated as the difference between the total Cr and Cr(VI) concentrations.

## 3. Results

### 3.1. Fabrication and Characterization of SMP-Fr-Py

The precise synthesis strategy of hyper-cross-linked polymers (HCPs) gives them more refined structural characteristics, making them promising for a wide range of applications. Various methods have been used to prepare knitted HCPs, including solvent knitting, knitting with FDA as the external cross-linking agent, and Scholl coupling reaction methods [40]. Knitting with FDA as the external cross-linking agent is the fastest method to generate polymer networks. In particular, the Scholl coupling reaction can facilitate the formation of braided fluorescent polymers due to the formation of C-C bonds directly between the aromatic units, which leads to an increase in the conjugated structure of the polymer and facilitates the transfer and transmission of electrons. Based on this property, we prepared SMP-Fr-Py using the Scholl coupling reaction, using ferrocene and pyrrole as building blocks (molar ratio 1:1) for the removal of Cr(VI) from water. A comparative adsorbent, HCP-Fr-Py, was prepared using an additive cross-linker FDA.

**Figure 1:**
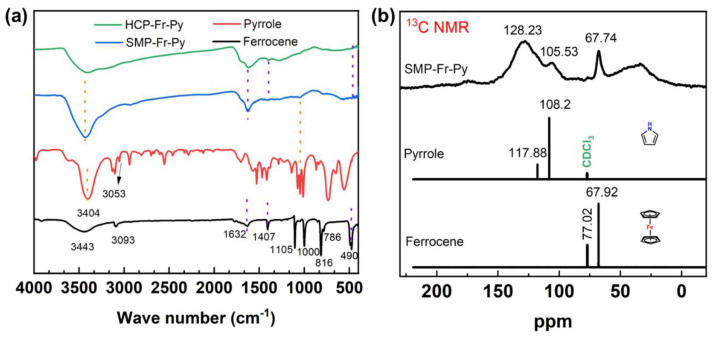
FTIR spectra of samples (**a**), and ^13^C CP-MAS NMR of SMP-Fr-Py (**b**).

In order to ascertain both the starting materials and the structural attributes of the polymers, FTIR spectra were recorded (Figure 1a). In the spectra, the peak observed at 3093 cm^−1^ was due to the stretching vibration of C-H on the cyclopentadienyl rings, whereas the bending vibration of C-H was discerned at a range of 1000–786 cm^−1^. The peaks noted at 1632 cm^−1^ and 1407 cm^−1^ corresponded to the stretching vibrations of the C=C cyclopentadienyl rings and the stretching vibration of C-Fe at 490 cm^−1^, respectively [37]. In addition, the characteristic peaks of pyrrole also appeared around 1660 cm^−1^ and 1200–950 cm^−1^. The relevant characteristic peaks can also be observed in the FTIR spectra of the polymers, indicating the successful binding of ferrocene and pyrrole into the polymer backbone. Solid-state ^13^C cross-polarization magic angle spinning nuclear magnetic resonance (CP-MAS NMR) spectroscopy detected signals at 108.2 and 117.88 ppm, which were assigned to the carbon atoms of the heteroaromatic ring [41]. The signals at approximately 67.92 ppm were assigned to the C=C cyclopentadienyl rings. Furthermore, the ^13^C CP-MAS NMR of SMP-Fr-Py revealed characteristic signals of pyrrole (128.23 ppm) and ferrocene (67.74 ppm). The peak around 128.23 ppm was broader and was assumed to contain the C-C bond formed after the Scholl coupling reaction. This indicates the successful copolymerization of ferrocene and pyrrole via the Scholl coupling reaction (Figure 1b).

**Figure 2:**
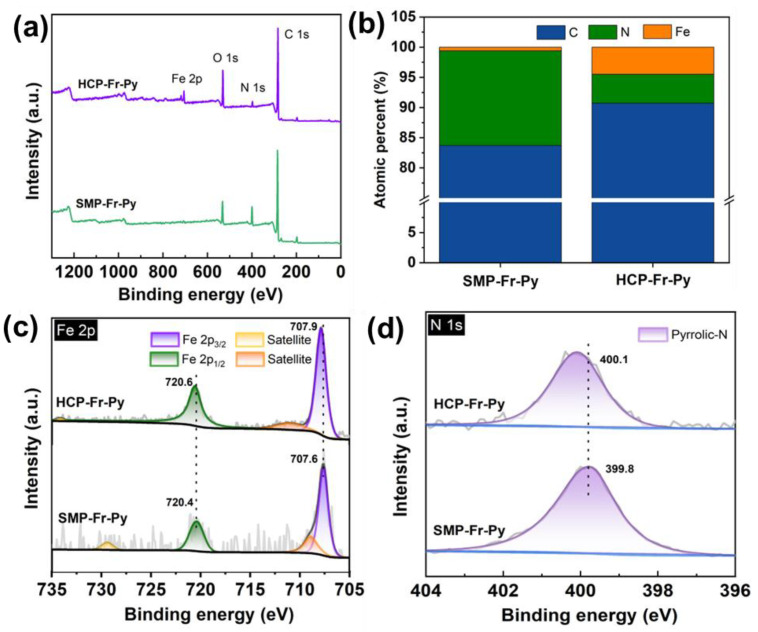
(**a**) XPS total survey spectra, (**b**) atomic composition, (**c**) high-resolution Fe 2p XPS spectra, and (**d**) N 1s XPS spectra of SMP-Fr-Py and HCP-Fr-Py.

The XPS is an excellent analytical tool for studying the chemical composition of material surfaces, and it can also be used to determine the elemental composition of prepared materials. Figure 2a shows the XPS total survey spectrums of SMP-Fr-Py and HCP-Fr-Py. The results show that the atomic percentages of the C, N, and Fe elements on the surface of the two materials differ significantly, with 83.7%, 15.7%, and 0.6%, and 90.7%, 4.8%, and 4.5%, respectively (Figure 2b). The Fe 2p binding energy peaks at 707.6 eV and 720.4 eV correspond to Fe^2+^ in ferrocene [34]. The change in binding energy when comparing SMP-Fr-Py and HCP-Fr-Py (Figure 2c,d) provided evidence of strong interactions between Fe and N species. The maximums of the Fe 2p1/2 and Fe 2p3/2 peaks shifted from 707.9 eV for HCP-Fr-Py to 707.6 eV for SMP-Fr-Py and 720.6 eV for HCP-Fr-Py to 720.4 eV for SMP-Fr-Py, respectively. At the same time, the maximum value of the N 1s peak also decreased from 400.1 to 399.8 eV. The simultaneous decrease in the binding energy of Fe 2p and N 1s in SMP-Fr-Py indicated low electron density in this polymer. The above results indicate that the physicochemical properties of the materials obtained through the two synthesis methods were significantly different. More importantly, it was extremely strongly suggested that the metallocene ring in ferrocene was directly linked to pyrrole through C-C bonding, which enhanced the mutual synergy between the groups and expanded the conjugated structure of the polymer backbone, which was favorable for electron transfer and transmission.

**Figure 3:**
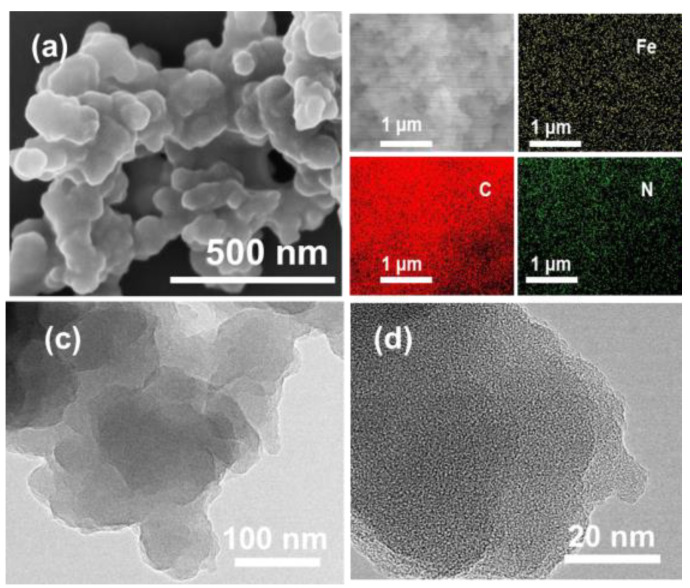
(**a**) SEM image and (**b**) the corresponding element mappings for C (red), Fe (yellow), and N (green). (**c**,**d**) TEM images of SMP-Fr-Py at different magnifications.

The surface morphology and porosity of the polymers were investigated using SEM and TEM. Figure 3a shows the SEM image of these networks, and the results show that the material exhibited an irregular blocky structure (particles larger than 100 nm) stacked layer by layer. The microporous structure was quite different compared to the ferrocene-based pore materials reported in some previous studies [42,43]. The energy dispersive X-ray spectroscopy (EDS) analysis of SMP-Fr-Py indicated the presence of C, N, and Fe. The corresponding elemental mapping of SEM-EDS (Figure 3b) illustrated a uniform distribution of these elemental species throughout the sample. The high-resolution TEM images revealed microchannels containing various microporous and mesoporous structures (Figure 3c), and SMP-Fr-Py appeared as a lamellar structure composed of conjugated aromatic rings (Figure 3d).

### 3.2. Cr(VI) Removal with Polymers

**Figure 4:**
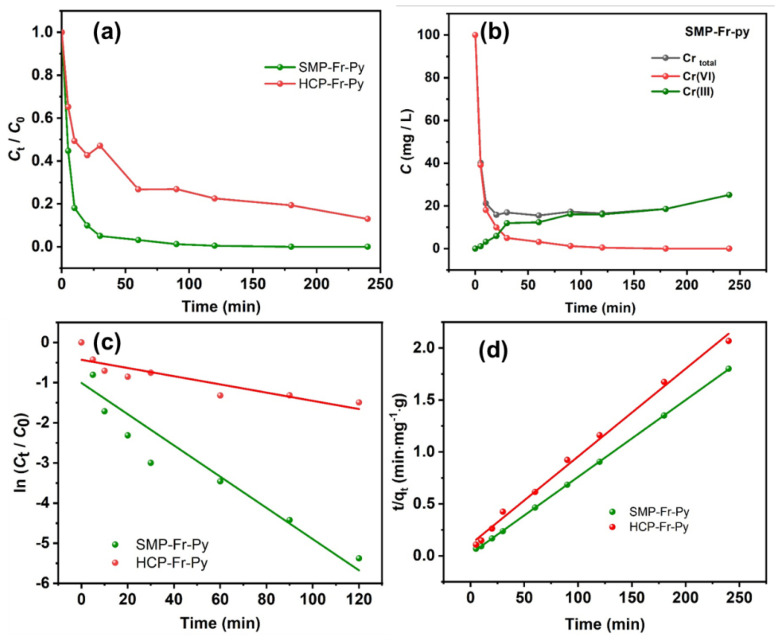
(**a**) The removal efficiency of Cr(VI) by SMP-Fr-Py and HCP-Fr-Py. (**b**) Changes in Cr total, Cr(VI), and Cr(III) concentrations at a fortification level of 100 mg/L of Cr(VI). The corresponding adsorption kinetics: pseudo-first-order (**c**), as well as pseudo-second-order kinetics models (**d**). Conditions: the initial solution pH = 2; m = 30 mg; V = 40 mL; c_0_ = 100 mg·L^−1^; T = 298 K.

In order to evaluate the effect of different synthesis methods on the removal efficiency of Cr(VI), batch-type Cr(VI) removal experiments were conducted under fixed conditions (the initial solution pH = 2; m = 30 mg; V = 40 mL; c_0_ = 100 mg·L^−1^; T = 298 K). The removal capacity of the adsorbent for Cr(VI) is shown in Figure 4a. The Fe-N conjugated organic polymer (SMP-Fr-Py) was prepared from ferrocene and pyrrole through a Scholl coupling reaction, which significantly improved the Cr(VI) removal performance compared with the polymer prepared by adding cross-linking agent FDA (HCP-Fr-Py). The experimental results showed that at a pH of 2 and a temperature of 25 °C, the removal of Cr(VI) reached 90% for SMP-Fr-Py and only 58% for SMP-Fr-Py after 20 min of reaction. Subsequently, 99% and 78% were achieved after 120 min of reaction, respectively.

The removal of Cr(VI) using heterogeneous materials as media involves direct solution reduction/precipitation and surface adsorption/reduction processes. Hence, for the first time, the concentrations of dissolved Cr(VI) and total Cr during the removal process were quantified [28]. The concentration of total Cr in the solution decreased from the initial value of 100.0 mg/L to 16.9 mg/L within 30 min (Figure 4b). This finding indicates that most dissolved Cr species were transferred to the surface of SMP-Fr-Py. Furthermore, the concentration of total Cr exceeded that of Cr(VI), which may be attributed to the production of dissolved Cr(III). The results confirm that the concentration of Cr(III) increased to 11.9 mg/L after 30 min of SMP-Fr-Py action, indicating the reduction of Cr(VI) by SMP-Fr-Py. However, during the reaction, the concentration of Cr(VI) decreased progressively, whereas the concentration of Cr(III) increased, resulting in a minimal value of total Cr concentration. Moreover, desorption of Cr(III) was observed during this process.

The adsorption data were further subjected to pseudo-first-order and pseudo-second-order kinetic simulations, as shown in Figure 4c,d. The results demonstrated that both materials exhibited better conformity with the pseudo-second-order kinetic model in terms of adsorption behavior. The correlation coefficients for the pseudo-second-order kinetic model were 0.999 and 0.994 for the two materials, respectively. The correlation coefficients for the pseudo-first-order kinetic model were 0.88 and 0.75 for the two materials, respectively. These findings suggest that the adsorption process was homogeneous [44].

**Figure 5:**
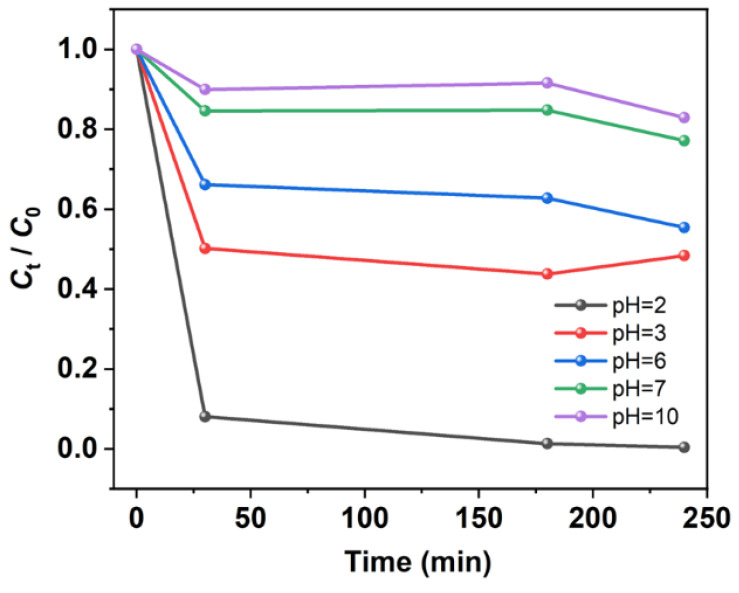
Effect of solution pH on Cr(VI) removal by SMP-Fr-Py (m = 30 mg; V = 40 mL; *C*_0_ = 100 mg·L^−1^; T = 298 K).

One of the most crucial factors in water treatment processes that affects adsorption is the pH of the wastewater. This is because the pH level of the solution has a strong impact not just on the surface chemistry of the adsorbent, but also on the polarity of the adsorbent–adsorbate–solvent interactions [7,45]. Overall, the oxidation state of Cr(VI) ions is greatly influenced by the pH level of the contaminant solution. In an acidic environment with a pH range of 1.0 to 5.0, HCrO_4_^−^ and Cr_2_O_7_^2−^ are the prevailing species, but under neutral and alkaline conditions (pH of 5.0 to 10.0), CrO_4_^2−^ prevails. Despite these varying conditions, the anionic nature of Cr(VI) remains largely unaffected [46]. Hence, it can be assumed that the adsorption efficiency of Cr(VI) removal is related to the charge properties of the adsorbent surface. Figure 5 illustrates the effect of solution pH on the removal of Cr(VI) by SMP-Fr-Py, revealing that the removal efficiency decreases as the solution pH increases. At a low pH, the nitrogen atoms in the polymer matrix protonate and attract Cr(VI) anions. However, as the pH increases from 2 to 10, the removal efficiency of Cr(VI) decreases due to the reduction in nitrogen atom protonation and the competition for adsorption sites on the SMP-Fr-Py surface between OH^-^ and Cr(VI) anions [13]. Additionally, in an acidic environment, Cr(VI) was highly oxidizing, and the presence of SMP-Fr-Py could increase the removal rate of Cr(VI) due to its ability to react with reducing substances (e.g., hydrogen radicals, ferrous ions, etc.) [3,25]. Therefore, SMP-Fr-Py demonstrates a strong potential to remove Cr(VI) under lower pH conditions due to these factors.

At a pH of 2.0 and an initial Cr(VI) concentration of 100 mg/L, we investigated how the removal of Cr(VI) was affected by different SMP-Fr-Py dosing amounts. Figure S2 shows that an increase in SMP-Fr-Py dosage from 10 mg to 40 mg during the first 5 min of the reaction resulted in an increase in Cr(VI) removal from 23.2% to 93.6%. However, the removal rate of Cr(VI) by 30 mg of SMP-Fr-Py was 7% lower than that by 40 mg of SMP-Fr-Py. Nonetheless, after 90 min of reaction, the removal rates for Cr(VI) by 30 mg and 40 mg of SMP-Fr-Py were comparable at 99.9%. At equilibrium, the active site availability of SMP-Fr-Py was illustrated by its ability to remove only 59.3% of Cr(VI) with a dosage of 10 mg.

**Figure 6:**
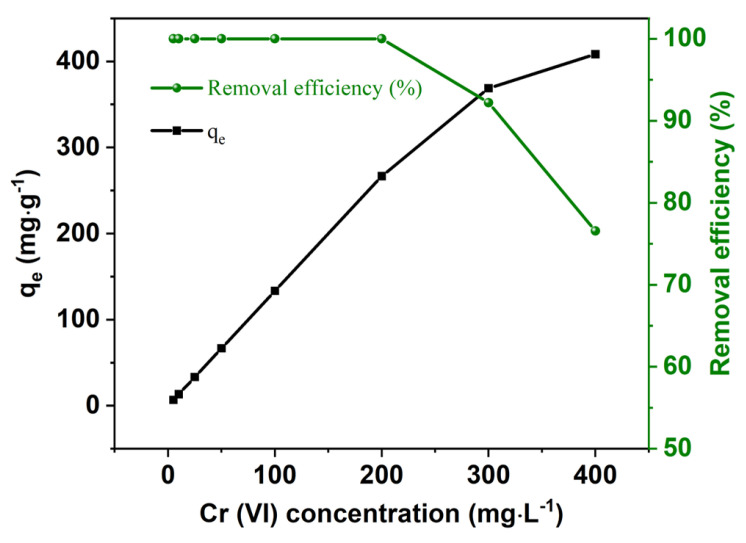
Effect of Cr(VI) concentration on Cr(VI) removal by SMP-Fr-Py (pH = 2; m = 30 mg; V = 40 mL; T = 298 K; reaction time: 720 min).

The effect of different initial Cr(VI) concentrations on the removal of Cr(VI) by SMP-Fr-Py was investigated under the following conditions: initial solution pH = 2, m = 30 mg, and V = 40 mL. As shown in Figure 6, almost all of the Cr(VI) was removed from the aqueous solution when the initial concentration of Cr(VI) was low (≤200 mg/L), but when the initial concentration of Cr(VI) was increased from 200 mg/L to 400 mg/L, the removal of Cr(VI) gradually decreased due to the limited availability of active sites on SMP-Fr-Py. Furthermore, the removal rate of Cr(VI) decreased from 99.9%. The Cr(VI) removal capacity of SMP-Fr-Py increased as the initial concentration of Cr(VI) increased. When the initial concentration of Cr(VI) was higher (200–400 mg/L), more Cr(VI) ions competed for the limited active sites on SMP-Fr-Py, which led to a gradual slowdown in the rate of increase of the removal capacity.

**Figure 7:**
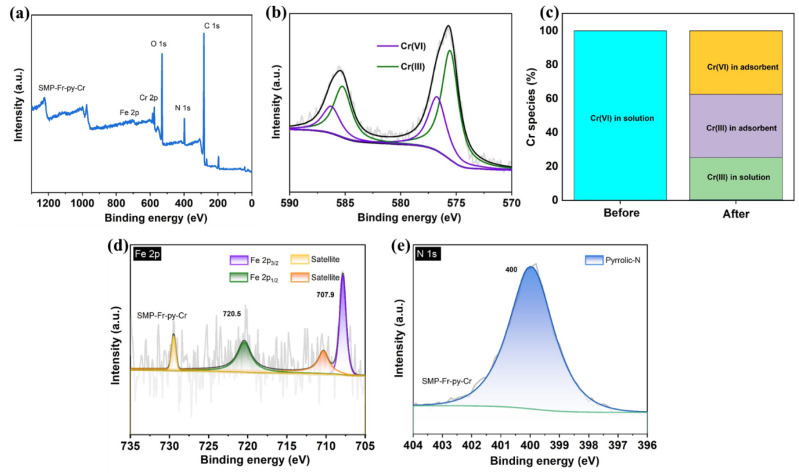
(**a**) Total survey spectra; (**b**) high-resolution Cr 2p spectrum; (**c**) Cr species changes before and after remediation with SMP-Fr-Py; (**d**) high-resolution Fe 2p spectra; and (**e**) N 1s spectra of SMP-Fr-Py-Cr.

When the effect of solution pH was investigated, the results showed that SMP-Fr-Py was more effective in removing Cr(VI) under lower pH conditions. In order to get an accurate understanding of the removal mechanism of Cr(VI) by SMP-Fr-Py, the Cr(VI) solution after adsorption by SMP-Fr-Py was analyzed using XPS and ICP-AES. XPS was used to discover the adsorbed Cr species and the surface functional groups of the adsorbent during the removal of Cr(VI). Figure 7a shows the total XPS spectrum of SMP-Fr-Py after the removal of Cr(VI). During the removal of Cr(VI), new peaks of 577 and 587 eV appeared, which were associated with the Cr 2p 3/2 and Cr 2p 1/2 orbitals, respectively. This indicated that Cr was successfully adsorbed on the surface of SMP-Fr-Py. Based on the high-resolution Cr 2p spectra (Figure 7b), it was confirmed that both the Cr 2p 3/2 and Cr 2p 1/2 regions were split into two specific Cr(VI) and Cr(III) regions. The binding energies of 587.5 and 578.5 eV were associated with Cr(VI) species, whereas the binding energies of 585.6 and 576.8 eV were associated with Cr(III). This indicates that Cr(VI) and Cr(III) coexist on the surface of SMP-Fr-Py. The detailed Cr 2p spectra showed that 35% of the Cr species were Cr(VI) and the remaining 65% were Cr(III). This indicates that a substantial quantity of Cr(VI) was converted to Cr(III) during the Cr(VI) removal process, and the resultant Cr(III) possibly adhered to the surface of SMP-Fr-Py.

### 3.3. Removal Mechanism

**Figure 8:**
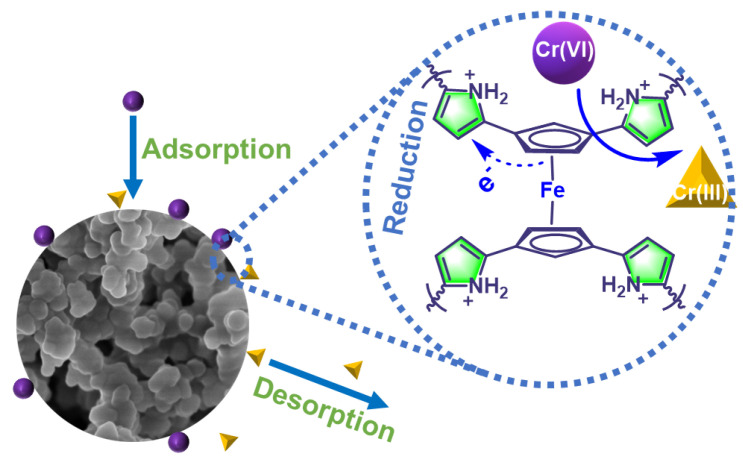
Schematic of the possible Cr(VI) remediation mechanism with SMP-Fr-Py.

The concentrations of Cr species in the Cr(VI) solution were measured using ICP and XPS data (Figure 7c) to evaluate the effectiveness of the removal process. The proposed restoration mechanism of Cr(VI) by SMP-Fr-Py is as follows: Under acidic conditions (pH = 2), the nitrogen atoms in the adsorbent can be protonated to form =NH_2_^+^ groups, facilitating the electrostatic attraction of Cr(VI) ions onto SMP-Fr-Py. Additionally, a redox reaction occurs between the secondary amine group of SMP-Fr-Py and the ferrous iron of ferrocene, resulting in the reduction of Cr(VI) to Cr(III) [47]. This reduction process causes Cr(III) species to be adsorbed on the surface of SMP-Fr-Py through intermolecular interactions. Afterwards, these species are released back into the solution. This mechanism is illustrated in Figure 8.

### 3.4. Stability Test

**Figure 9:**
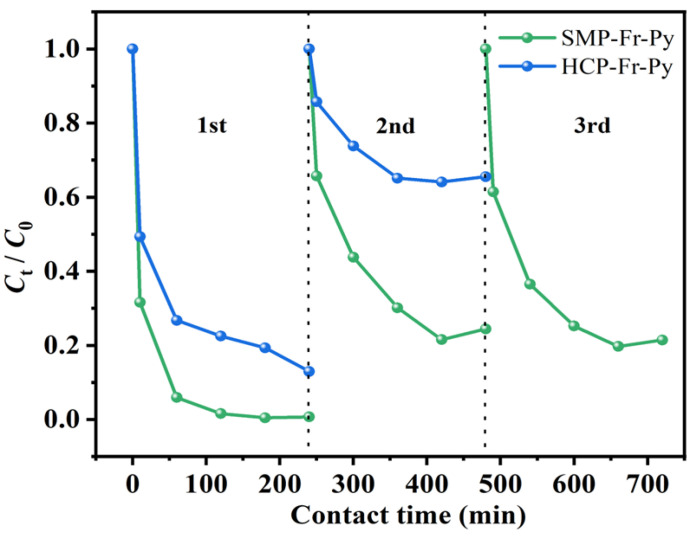
Stability tests for Cr(VI) removal by SMP-Fr-Py and HCP-Fr-Py.

The stability of this material is a key issue for its practical application. Therefore, the SMP-Fr-Py that was used was separated from the suspension and reused for Cr(VI) removal under the same conditions. Compared with HCP-Fr-Py, the stability of SMP-Fr-Py was significantly better, and after three cycles, SMP-Fr-Py still showed good Cr(VI) removal. However, the total removal of Cr(VI) by SMP-Fr-Py decreased from 99% to 75.6% and 78.6% in the second and third cycles, respectively (Figure 9). In contrast, Cr(VI) removal by HCP-Fr-Py in the second cycle was only 35%. The gradual decrease in Cr(VI) removal by SMP-Fr-Py may be due to its surface deactivation, which reflects the observed changes in the binding energies of Fe and N in the XPS analysis (Figure 7d,e). Thus, the better performance of SMP-Fr-Py for the removal of Cr(VI) can also be attributed when compared to HCP-Fr-Py.

The pore structure of SMP-Fr-Py was characterized using nitrogen adsorption/desorption isotherms at 77 K. The measurements obtained from the nitrogen adsorption/desorption isotherms allowed for determination of the pore structure of SMP-Fr-Py. Initially, the specific surface area was found to be 127.2 m^2^/g. However, after conducting experiments to remove Cr(VI), the specific surface area decreased to 93.1 m^2^/g. This decrease in specific surface area could be attributed to the adsorption of Cr species by the material, as observed in the type IV adsorption isotherm depicted in Figure S1. In conclusion, these findings suggest that the SMP-Fr-Py material demonstrates the capability of physically adsorbing Cr species. Furthermore, SMP-Fr-Py demonstrated a maximum Cr(VI) removal of 408 mg/g at a temperature of 25 °C and a pH of 2, surpassing the performance of numerous comparable materials documented in the literature (refer to Table 1).

Lastly, the SEM image and IR spectra of SMP-Fr-Py were examined before and after the reaction, as depicted in Figure S3. It was observed that the microstructure of the material experienced alterations and the surface functional groups were transformed following the reaction, potentially resulting in a reduction in the removal efficiency of the material.

## 4. Conclusions

A Fe-N conjugated organic polymer was successfully prepared using a Scholl coupling reaction and was applied to the remediation of Cr(VI) wastewater. The ability of SMP-Fr-Py to remove Cr(VI) was affected by the solution pH, the amount of SMP-Fr-Py, and the initial concentration of Cr(VI). The kinetic data exhibited good agreement with the pseudo-second-order kinetics model. The direct linking of ferrocene and pyrrole through C-C bonding increased the conjugated structure of the polymer, which facilitated electron transport and transfer, significantly improving the removal performance of Cr(VI) compared with HCP-Fr-Py. In addition, the characterization of the reaction complexes and measurements of Cr species conversion indicated that the removal of Cr(VI) was accompanied by several processes, including adsorption, reduction, and desorption. The mechanistic analysis suggests that the synergistic effect of Fe-N is responsible for the excellent performance of SMP-Fr-Py. In conclusion, SMP-Fr-Py exhibits a good ability to remove and recover Cr(VI) and is considered a potential material for the treatment of Cr(VI)-contaminated wastewater.

## Figures and Tables

**Table 1:** Comparison of the Cr(VI) removal capacity of Fe-based materials.

Adsorbent	Cr(VI) Removal Capacity (mg/g)	Reference
FeS/BNC	211.3	[23]
TP-NZVI-OB (2:1)	170	[30]
nZVI/BC/CA	86.4	[48]
Fe/PBC-ND	25.7	[49]
NZVI-SDBC	64.1	[17]
SBC-nZVI	84.4	[50]
rGO-nZVI	50.1	[51]
nZVI@HCl-BC	18.8	[3]
SMP-Fr-Py	408	This work

## Data Availability

Not applicable.

## Supplementary Materials

The following supporting information can be downloaded at: https://www.mdpi.com/article/10.3390/polym15132918/s1, Figure S1: The nitrogen adsorption/desorption isotherms (a) and the pore size distribution (b) of SMP-Fr-Py, Figure S2: Effect of SMP-Fr-Py dosage on Cr(VI) removal (pH = 2; V = 40 mL; T = 298 K); Figure S3. SEM image of SMP-Fr-Py after reaction (a) and comparison of FTIR spectra before and after the reaction.
