# Supplementary material for: Fe-N-Doped Conjugated Organic Polymer Efficiently Enhanced the Removal Rate of Cr(VI) from Water
Source: Polymers (Basel). 2023 Jun 30;15(13):2918. doi: 10.3390/polym15132918 (PMC10346507; doi:10.3390/polym15132918)
Supplement: Supplementary file 1 [file polymers-15-02918-s001.zip › polymers-2423867-supplementary.pdf]

Supporting Information

Fe-N doped Conjugated Organic Polymer Efficiently Enhanced the Removal rate of Cr (VI) from Water

*Cheng Tang, Tao Hu, Chengzhen Du, Ziqin Liao, Wenyan Cheng, Fen Wang, Xiaoli Hu[*], Kunpeng Song**

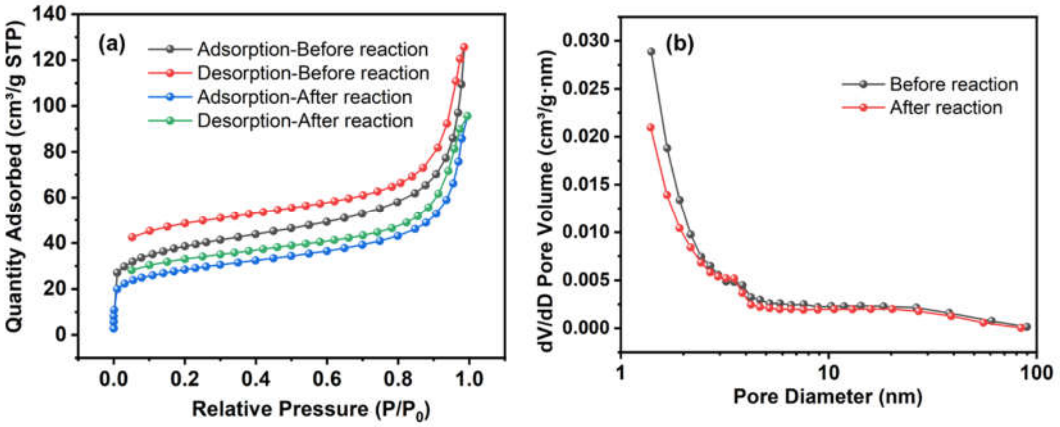

Figure S1. The nitrogen adsorption/desorption isotherms (a) and the pore size distribution (b) of SMP-Fr-Py (before reaction and after reaction).

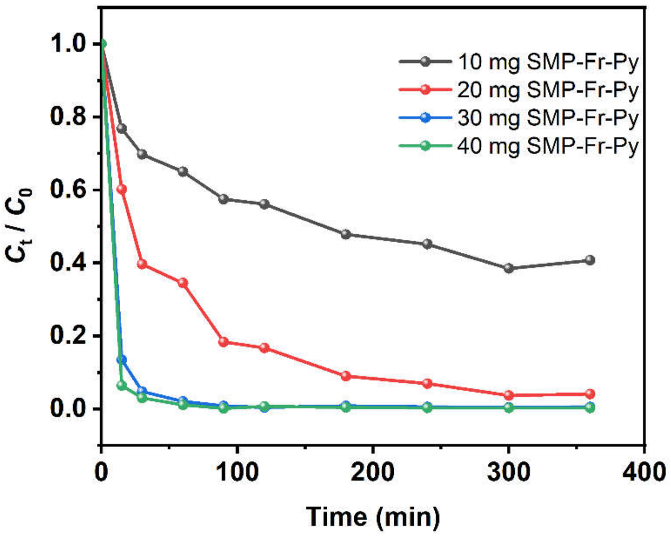

Figure S2. Effect of SMP-Fr-Py dosage on Cr(VI) removal. (pH=2; V=40 mL; T=298 K)

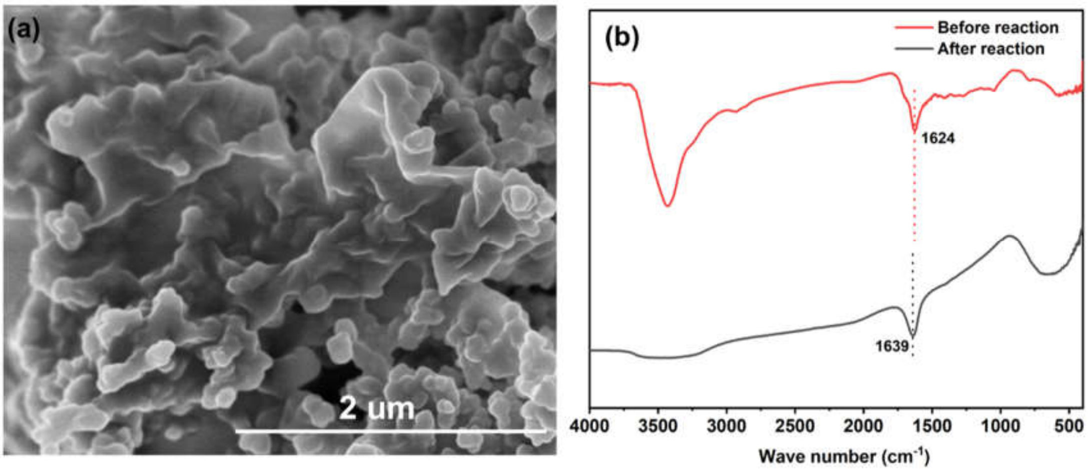

Figure S3. SEM image of SMP-Fr-Py after reaction (a) and comparison of FTIR spectra before and after the reaction.
